# Fluttering cord-like thrombus in the aortic arch

**DOI:** 10.1186/s13019-022-01843-5

**Published:** 2022-05-03

**Authors:** Yuki Kuroda, Akira Marui, Yoshio Arai, Atsushi Nagasawa, Shinichi Tsumaru, Ryoko Arakaki, Jun Iida, Yuki Wada, Yoshiharu Soga

**Affiliations:** Department of Cardiovascular Surgery, Kokura Memorial Hospital, 3-2-1 Asano Kokurakita-ku, Kitakyushu-shi, Fukuoka 802-8555 Japan

**Keywords:** Aortic thrombus, Cord-like thrombus, Hypothermic circulatory arrest, Carotid artery, Case report

## Abstract

**Background:**

The optimal treatment for aortic thrombus remains to be determined, but surgical treatment is indicated when there is a risk for thromboembolism.

**Case presentation:**

A 47-year-old male presented with weakness in his left arm upon awakening. Contrast-enhanced computed tomography and transesophageal echocardiography revealed a mobile pedunculated object suggestive of a thrombus arising from the ascending aorta and extending to the left common carotid artery. It was removed under hypothermic circulatory arrest and direct cannulation of the left carotid artery to avoid carotid thromboembolism. Histopathological examination revealed that the object was a thrombus. The patient had an uneventful postoperative course and was discharged 9 days after surgery.

**Conclusion:**

When a thrombus in the aortic arch extends to the neck arteries, direct cannulation of the neck arteries with selective cerebral perfusion via cervical incision is a useful technique.

**Supplementary Information:**

The online version contains supplementary material available at 10.1186/s13019-022-01843-5.

## Background

A fluttering cord-like thrombus in the aortic arch is occasionally observed in clinical practice. The pathophysiology of these lesions is unclear, however, and the optimal management is still under debate. Emergent surgical removal is necessary when the risk for embolism is high. Here, we report a useful strategy for a fluttering cord-like thrombus in the aortic arch extending to the left carotid artery.

## Case presentation

**Fig. 1:**
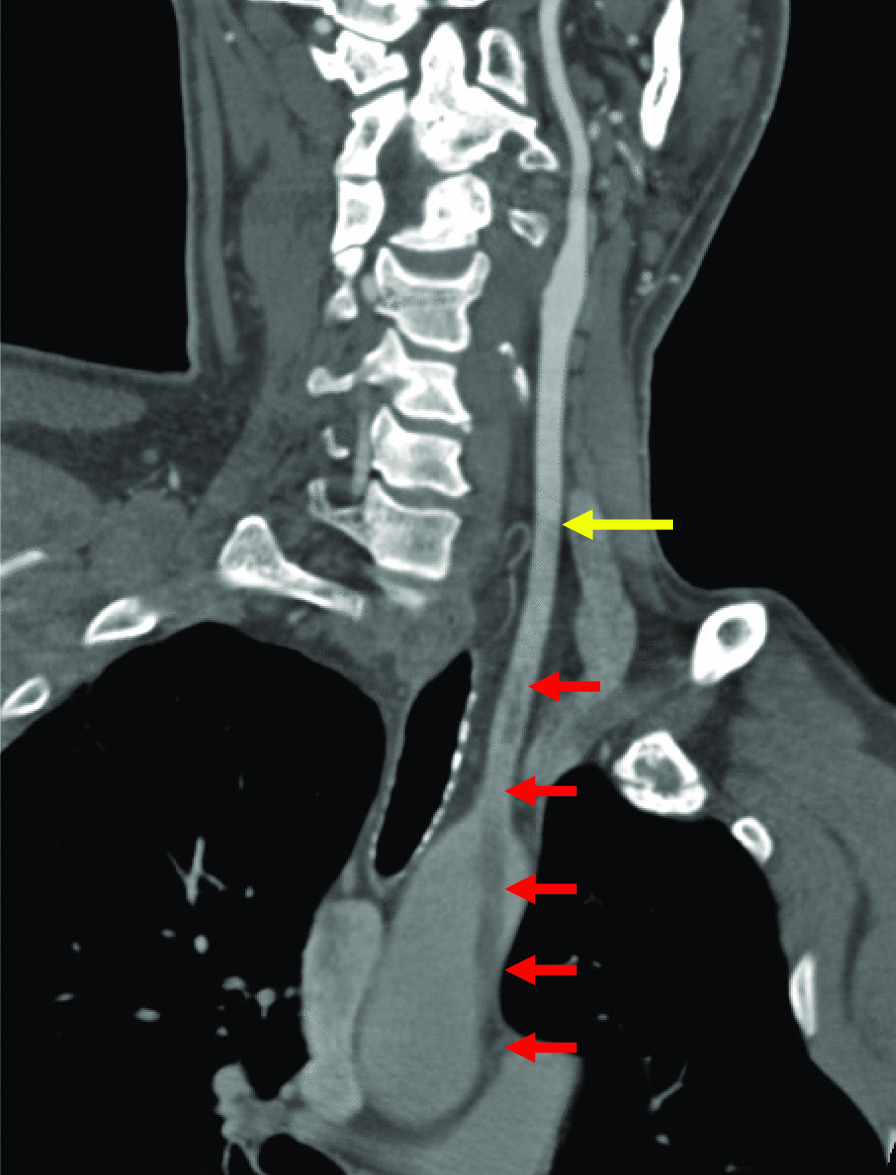
Contrast-enhanced sagittal oblique view of the thoracic aorta showing intraluminal, hypodense, and linear strands of a non-enhancing structure (red arrows) in the ascending aorta extending to the left common carotid artery (yellow arrow), suggestive of a thrombus

**Fig. 2:**
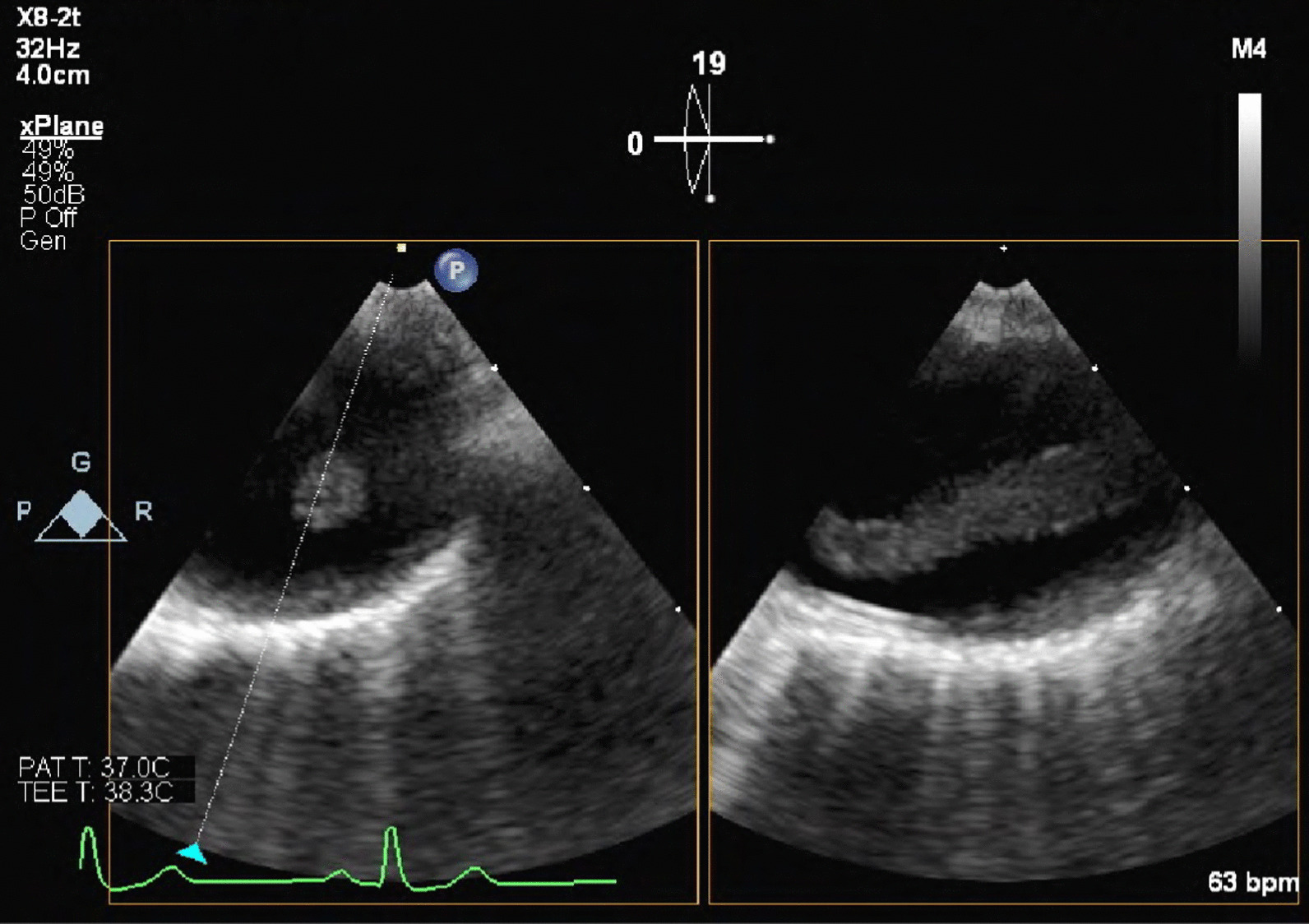
Transesophageal echocardiography showing a fluttering cord-like thrombus in the aortic arch extending to the left common carotid artery

A 47-year-old male presented with weakness in his left arm upon awakening. Mild fine motor impairment and mild paresthesia were observed. Magnetic resonance imaging (MRI) showed cerebral infarction in the left frontal and parietal lobes. Contrast-enhanced computed tomography (CT) revealed a well-defined pedunculated cord-like object in the aortic arch extending from the lesser curvature of the ascending aorta into one-third of the length of the left common carotid artery (Fig. 1). CT also showed mural thrombus and stenosis of the abdominal aorta and obstruction of the right common iliac artery and the left deep femoral artery. Transesophageal echocardiography revealed a mobile pedunculated object suggestive of a thrombus arising from the ascending aorta and extending to the left common carotid artery (Fig. 2, Additional file 1). Hematologic investigations, including lipid profile, hematocrit, platelet count, protein C, protein S, and antiphospholipid antibody, were unremarkable.

Because the object was considered high risk for additional embolic events, the patient underwent emergency surgery. The operation was performed through a median sternotomy. Cardiopulmonary bypass was established by cannulation of the right femoral artery and right atrium. In order to prevent embolism of the thrombus, we undertook direct cannulation into the left common carotid artery distal to the thrombus. The left common carotid artery was exposed by a separate left cervical incision (parallel to the left sternocleidomastoid muscle). While cooling the patient to 28 °C (measured by bladder probe), the left common carotid artery was incised and directly cannulated with a balloon-tipped catheter for selective antegrade cerebral perfusion (SACP) via the left cervical incision. The proximal side of the left common carotid artery was clamped. Under hypothermic circulatory arrest, the ascending aorta was opened through a longitudinal incision. The cord-like object suggestive of a thrombus was attached to the lesser curvature of the ascending aorta and extended into the left common carotid artery. The thrombus was easily removed from the aortic wall. The brachiocephalic artery was cannulated with a balloon-tipped catheter for SACP. Thrombectomy with a 5 Fr Fogarty catheter was performed into the left common carotid artery, but no thrombus remained. The aortotomy was closed with 4–0 polypropylene continuous suture.

**Fig. 3:**
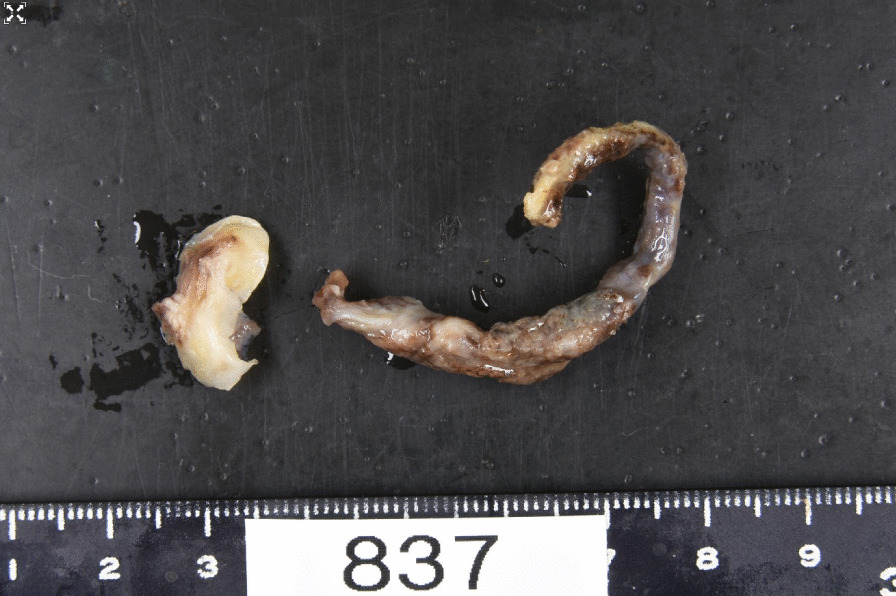
Specimens of the excised thrombus

Histopathological examination revealed that the object was a thrombus (Fig. 3). The postoperative course was uneventful. No additional embolism was observed. The patient was discharged 9 days after surgery. The patient was treated with oral aspirin, clopidogrel, and warfarin postoperatively. No recurrence of the thrombus was observed at the one-year follow-up.

## Discussion and conclusions

The pathophysiology of a fluttering cord-like thrombus in the aortic arch is unclear. Thrombophilic states are not always observed in patients with thrombus in the aortic arch. Laperche et al. reported that, among 23 patients with mobile thrombi of the aortic arch, only 4 cases presented with thrombophilic states [1]. In our case, the coagulation test did not reveal any coagulopathy. However, mural thrombus and stenosis of the abdominal aorta and obstruction of the right common iliac artery and the left deep femoral artery suggest some kind of thrombophilic state.

Evidence related to management of thrombus in the aorta is very limited. A few teams have reported successful management with anticoagulant therapy [2, 3]. Pharmacological treatment (heparinization), endovascular stenting [4], and surgery have been proposed. Although no comparative data are available, pharmacological treatment is indicated when the risk of thromboembolism is considered to be low. Endovascular stent graft exclusion sometimes carries the risk of procedure-related embolism, especially when the thrombus extends to branches. In our case, because MRI revealed cerebral infarction and the fluttering cord-like thrombus extended to the left common carotid artery, the thrombus was considered high risk for additional cerebral infarction, and we performed thrombectomy.

Traditionally, aortic thrombi have been removed under hypothermic circulatory arrest either by distal ascending aortic cannulation [5] or femoral artery cannulation [6]. In the present case, we considered using axillary artery perfusion in order to prevent embolism caused by retrograde perfusion because the patient had mural thrombi in the abdominal aorta and the iliac artery. However, the axillary arteries were small and inappropriate for perfusion, so we used femoral artery perfusion. Fortunately, procedure-related embolism did not occur.

Kalangos et al. reported the successful removal of a thrombus in the proximal ascending aorta without hypothermic circulatory arrest [7]. In this case, because the thrombus extended to the aortic arch, we performed thrombectomy under hypothermic circulatory arrest and selective cerebral perfusion. To prevent distal embolization of the thrombus, we used direct cannulation of the left common carotid artery with clamping at the proximal side. With SACP for brain protection, we were able to remove the thrombus safely and reliably. This technique is considered useful when a thrombus in the aortic arch extends to the neck arteries. In order to use this technique, it is also important to check the location of the thrombus with preoperative CT and carotid ultrasound.

A fluttering cord-like thrombus in the aortic arch may develop in patients who do not have obvious coagulopathy. When a thrombus in the aortic arch extends to the neck arteries, direct cannulation of the neck arteries with selective cerebral perfusion via cervical incision is a useful technique.

## Supplementary Information

**Additional file 1**. Transesophageal echocardiography showing a fluttering cord-like thrombus in the aortic arch extending to the left common carotid artery.

## Data Availability

The data that support the findings are available from the corresponding author on reasonable request.

## Abbreviations

MRI: Magnetic resonance imaging
CT: Computed tomography
SACP: Selective antegrade cerebral perfusion
